# Supplementary material for: Interventions to improve access to opioid agonist therapy in acute hospitals: A scoping review
Source: PLOS Ment Health. 2025 May 30;2(5):e0000322. doi: 10.1371/journal.pmen.0000322 (PMC12798493; doi:10.1371/journal.pmen.0000322)
Supplement: S1 Text — (PDF) [file pmen.0000322.s001.pdf]

S1_Text: Supplementary information

S1_Text: Search terms 2

S1_Text: Data charting tool 4

S1_Text: PRISMA-ScR checklist 9

S1_Text: Changes from review protocol 11

S1_Text: References 12

S1_Text: Search terms

Ovid MEDLINE(R) ALL: 1946 to July 29, 2024

1	opioid agonist therapy.ti,ab.
2	opioid agonist treatment.ti,ab.
3	opiate substitution therapy.ti,ab.
4	methadone.ti,ab.
5	buprenorphine.ti,ab.
6	medication assisted treatment.ti,ab.
7	medications for addiction treatment.ti,ab.
8	medication for opioid use disorder.ti,ab.
9	or/1-8
10	hospital.ti,ab.
11	inpatient.ti,ab.
12	emergency department.ti,ab.
13	accident and emergency.ti,ab.
14	admission.ti,ab.
15	surg$.ti,ab.
16	or/10-15
17	9 and 16
18	9 and (or/10-14)

Embase <1980 to 2022 Week 11>

1	opioid agonist therapy.ti,ab.
2	opioid agonist treatment.ti,ab.
3	opiate substitution therapy.ti,ab.
4	methadone.ti,ab.
5	buprenorphine.ti,ab.
6	medication assisted treatment.ti,ab.
7	medications for addiction treatment.ti,ab.
8	medication for opioid use disorder.ti,ab.
9	or/1-8
10	hospital.ti,ab.
11	inpatient.ti,ab.
12	emergency department.ti,ab.
13	accident and emergency.ti,ab.
14	admission.ti,ab.
15	surg$.ti,ab.
16	or/10-15
17	9 and 16
18	9 and (or/10-14)

APA PsychInfo <1806 to March Week 3 2022>

1	opioid agonist therapy.ti,ab.
2	opioid agonist treatment.ti,ab.
3	opiate substitution therapy.ti,ab.
4	methadone.ti,ab.
5	buprenorphine.ti,ab.
6	medication assisted treatment.ti,ab.
7	medications for addiction treatment.ti,ab.

8	medication for opioid use disorder.ti,ab.
9	or/1-8
10	hospital.ti,ab.
11	inpatient.ti,ab.
12	emergency department.ti,ab.
13	accident and emergency.ti,ab.
14	admission.ti,ab.
15	surg$.ti,ab.
16	or/10-15
17	9 and 16
18	9 and (or/10-14)

CINAHL <1961 to Week 11 2022>

1	opioid agonist therapy
2	opioid agonist treatment
3	opiate substitution therapy
4	methadone
5	buprenorphine
6	medication assisted treatment
7	medications for addiction treatment
8	medication for opioid use disorder
9	or/1-8
10	hospital
11	inpatient
12	emergency department
13	accident and emergency
14	admission
15	surg#
16	or/10-15
17	9 and 16
18	9 and (or/10-14)

S1_Text: Data charting tool

This form was implemented in Google Forms.

* indicates required question

Study details

1. Your name* ________________________
2. Study ID* ________________________
3. Study title* ________________________
4. Include?*
    a. Yes -> *Go to 7*
    b. No -> *Go to 5*

Exclusion reason

5. Exclusion reason* (select the first relevant listed reason)
    a. Not available in English
    b. Duplicate of study already included
    c. Conference/meeting abstract only
    d. Wrong study design (eg. case series <10, editorial, commentary, non-systematic review, guidance)
    e. No intervention to improve OAT
    f. Wrong population - not acute hospital patients (eg. outpatients, study in mental health hospital); participants do not use illicit opioids (eg. participants use prescription opioids)
    g. OAT is not dispensed in an acute hospital (eg. only dispensed after discharge)
    h. No relevant outcome (does not consider process-level outcomes, ie. improvement to OAT; or patient-level outcomes, eg. DAMA or readmission)
6. Notes on inclusion/exclusion decision: ________________________

End of data capture

Mixed Methods Appraisal Tool: screening questions

7. MMAT S1: Are there clear research questions?*
    a. Yes

b. No

8. MMAT S2: Do the collected data allow you to address the research questions?*

   a. Yes

   b. No

9. Notes for screening questions: ________________________

10. Did the study pass the screening questions?* (S1 and S2 should both be 'yes')

    a. Yes -> *Go to 11*

    b. No (no further data will be captured) *End of data capture*

Systematic reviews

11. Is the study a systematic review?*

    a. Yes -> *Go to 29*

    b. No -> *Go to 12*

Mixed Methods Appraisal Tool: full assessment

User guide: http://mixedmethodsappraisaltoolpublic.pbworks.com/w/file/fetch/127916259/MMAT_2018_criteria-manual_2018-08-01_ENG.pdf

12. Type of study*

    a. Qualitative

    b. Quantitative RCT

    c. Quantitative non-randomised

    d. Quantitative descriptive

    e. Mixed methods

13. MMAT assessment – Q1-Q5 relate to the type of study selected*

Qualitative
Q1. Is the qualitative approach appropriate to answer the research question?
Q2. Are the qualitative data collection methods adequate to address the research question?
Q3. Are the findings adequately derived from the data?
Q4. Is the interpretation of results sufficiently substantiated by data?
Q5. Is there coherence between qualitative data sources, collection, analysis and interpretation?

Quantitative randomized controlled trials
Q1. Is randomization appropriately performed?
Q2. Are the groups comparable at baseline?
Q3.Are there complete outcome data?
Q4.Are outcome assessors blinded to the intervention provided?
Q5 Did the participants adhere to the assigned intervention?

Quantitative nonrandomized

Q1. Are the participants representative of the target population?
Q2. Are measurements appropriate regarding both the outcome and intervention (or exposure)?
Q3. Are there complete outcome data?
Q4. Are the confounders accounted for in the design and analysis?
Q5. During the study period, is the intervention administered (or exposure occurred) as intended?

Quantitative descriptive
Q1. Is the sampling strategy relevant to address the research question?
Q2. Is the sample representative of the target population?
Q3. Are the measurements appropriate?
Q4. Is the risk of nonresponse bias low?
Q5. Is the statistical analysis appropriate to answer the research question?

Mixed methods
Q1. Is there an adequate rationale for using a mixed methods design to address the research question?
Q2. Are the different components of the study effectively integrated to answer the research question?
Q3. Are the outputs of the integration of qualitative and quantitative components adequately interpreted?
Q4. Are divergences and inconsistencies between quantitative and qualitative results adequately addressed?
Q5. Do the different components of the study adhere to the quality criteria of each tradition of the methods involved?

	Yes	No	Can't tell
Q1			
Q2			
Q3			
Q4			
Q5			

14. MMAT comments: _______________________

Intervention details

15. Country of intervention*: _______________________

16. Region/city of intervention: _______________________

17. Location of intervention* (eg. A&E, medical wards, etc.): _______________________

18. OAT medication* (select all that apply)

    a. Methadone

    b. Buprenorphine

    c. Other: _______________________

19. Years when intervention was implemented*: _______________________

20. Intervention elements:* (select all that apply)

    a. Measures to improve continuity after initiation of OAT in hospital (e.g. bridge prescriptions, partnerships with community OAT providers)

    b. Training and education in relation to OAT, including x-waiver training in the United States

    c. Multidisciplinary patient review

    d. OAT guidance or protocol

e. Providing advice about OAT to primary medical teams

f. Peer support for patients who need OAT

g. Electronic workflow that prompts staff to screen patients or supports other aspects of OAT

h. Multidisciplinary patient review, in which professionals from different backgrounds make decisions or provide recommendations in relation to OAT

i. Improving specialty-specific care for patients who are dependent on opioids

j. Measures to improve continuity of pre-existing OAT (e.g. medicine reconciliation)

21. Description of intervention:* ______________________

Study design

22. Population / participants*: ______________________

23. Does the study focus on a subgroup of patients? (If not, leave blank)

    a. Pregnant

    b. Breastfeeding

    c. Need pain relief

    d. Preparing for surgery or recovering from surgery

    e. May have unclear tolerance (for example due to recent opioid 'detoxification')

    f. Other: ______________________

24. Sample size: ______________________

25. Age (report whatever is provided, eg. "Median 45 (IQR 40-50"; "Mean 30 (sd 5)"; "Majority aged 15-45"; etc. If age groups are provided, include the number in each age group and we will estimate the mean): ______________________

26. Comparison group: ______________________

27. Outcomes: ______________________

28. Study design*: (select one)

    a. Qualitative

    b. Quantitative: cross-sectional (comparing intervention and control groups at the same point in time)

    c. Quantitative: cohort (following-up intervention and control groups)

    d. Quantitative: case control (recruiting participants with and without an outcome, eg. discharge against medical advice)

e. Quantitative: RCT

f. Quantitative: case series (every participant has the intervention, and the study describes participant characteristics)

g. Quantitative: before/after comparison (comparing outcomes before and after the intervention, either in the same sample or in different samples)

h. Other: ______________________

Findings

29. Brief description of study findings*: ______________________

30. Study concludes that the intervention improves OAT in acute hospitals*

    a. Yes

    b. No

    c. Unclear

Forwards and backwards reference search

31. Backward reference search (list references in the paper that look like they might be relevant, based on the title): ______________________

32. Forward reference search (list potentially relevant papers that have referenced this paper, using Google Scholar): ______________________

S1_Text: PRISMA-ScR checklist

Preferred Reporting Items for Systematic reviews and Meta-Analyses extension for Scoping Reviews (PRISMA-ScR) Checklist[1]

SECTION	ITEM	PRISMA-ScR CHECKLIST ITEM	REPORTED ON PAGE #
TITLE			
Title	1	Identify the report as a scoping review.	Title page; Abstract; Introduction/paragraph 3; Methods/protocol
ABSTRACT			
Structured summary	2	Provide a structured summary that includes (as applicable): background, objectives, eligibility criteria, sources of evidence, charting methods, results, and conclusions that relate to the review questions and objectives.	Abstract
INTRODUCTION			
Rationale	3	Describe the rationale for the review in the context of what is already known. Explain why the review questions/objectives lend themselves to a scoping review approach.	Introduction/paragraphs 1-3
Objectives	4	Provide an explicit statement of the questions and objectives being addressed with reference to their key elements (e.g., population or participants, concepts, and context) or other relevant key elements used to conceptualize the review questions and/or objectives.	Introduction/paragraph 3
METHODS			
Protocol and registration	5	Indicate whether a review protocol exists; state if and where it can be accessed (e.g., a Web address); and if available, provide registration information, including the registration number.	Methods/protocol
Eligibility criteria	6	Specify characteristics of the sources of evidence used as eligibility criteria (e.g., years considered, language, and publication status), and provide a rationale.	Methods/inclusion and exclusion criteria
Information sources*	7	Describe all information sources in the search (e.g., databases with dates of coverage and contact with authors to identify additional sources), as well as the date the most recent search was executed.	Methods/search strategy
Search	8	Present the full electronic search strategy for at least 1 database, including any limits used, such that it could be repeated.	Methods/search strategy; Supplementary Material/search terms
Selection of sources of evidence†	9	State the process for selecting sources of evidence (i.e., screening and eligibility) included in the scoping review.	Methods/screening and data charting
Data charting process‡	10	Describe the methods of charting data from the included sources of evidence (e.g., calibrated forms or forms that have been tested by the team before their use, and whether data charting was done independently or in duplicate) and any processes for obtaining and confirming data from investigators.	Methods/screening and data charting; Supplementary Material/data charting tool
Data items	11	List and define all variables for which data were sought and any assumptions and simplifications made.	Methods/screening and data charting; Supplementary Material/data charting tool

SECTION	ITEM	PRISMA-ScR CHECKLIST ITEM	REPORTED ON PAGE #
Critical appraisal of individual sources of evidence	12	If done, provide a rationale for conducting a critical appraisal of included sources of evidence; describe the methods used and how this information was used in any data synthesis (if appropriate).	Methods/quality assessment
Synthesis of results	13	Describe the methods of handling and summarizing the data that were charted.	Methods/synthesis and classification of interventions
RESULTS			
Selection of sources of evidence	14	Give numbers of sources of evidence screened, assessed for eligibility, and included in the review, with reasons for exclusions at each stage, ideally using a flow diagram.	Results /search results; Methods/Figure 1
Characteristics of sources of evidence	15	For each source of evidence, present characteristics for which data were charted and provide the citations.	Results/study characteristics; Supplementary Material/full data set
Critical appraisal within sources of evidence	16	If done, present data on critical appraisal of included sources of evidence (see item 12).	Results /study design and quality assessment
Results of individual sources of evidence	17	For each included source of evidence, present the relevant data that were charted that relate to the review questions and objectives.	Supplementary Material/full data set
Synthesis of results	18	Summarize and/or present the charting results as they relate to the review questions and objectives.	Results/intervention complements; Results/intervention classes
DISCUSSION			
Summary of evidence	19	Summarize the main results (including an overview of concepts, themes, and types of evidence available), link to the review questions and objectives, and consider the relevance to key groups.	Discussion/paragraphs 1-6
Limitations	20	Discuss the limitations of the scoping review process.	Discussion/limitations of the review
Conclusions	21	Provide a general interpretation of the results with respect to the review questions and objectives, as well as potential implications and/or next steps.	Discussion/conclusion
FUNDING			
Funding	22	Describe sources of funding for the included sources of evidence, as well as sources of funding for the scoping review. Describe the role of the funders of the scoping review.	Additional information/funding information

S1_Text: Changes from review protocol

We made three changes from our protocol:[2]

1. Our protocol stated that we would include relevant systematic reviews. We identified four relevant systematic reviews. We decided to exclude these reviews and focus on primary evaluations. The four systematic reviews are Weimer 2019,[3] Kaczorowski 2020,[4] French 2021,[5] and Bahji 2023.[6] These reviews are summarised in the discussion section of the main report. We included these reviews in backwards and forwards reference searching.

2. Clarified exclusion criteria to exclude studies that (1) were based on samples in which the majority of participants were not patients or staff at acute hospitals; (2) did not describe the key features of the intervention.

S1_Text: References

1. Tricco AC, Lillie E, Zarin W, O'Brien KK, Colquhoun H, Levac D, et al. PRISMA Extension for Scoping Reviews (PRISMA-ScR): Checklist and Explanation. Ann Intern Med. 2018;169: 467–473. doi:10.7326/M18-0850

2. Lewer D, Harris M, Scott J, Hope V, Brothers T, Holland A, et al. PROTOCOL: Opioid agonist therapy for management of opioid dependence in acute hospital settings: a systematic review and narrative synthesis. PROSPERO; 2022. Available: https://www.crd.york.ac.uk/prospero/display_record.php?RecordID=313237

3. Weimer M. Treatment of Opioid Use Disorder in the Acute Hospital Setting: a Critical Review of the Literature (2014–2019). Curr Addict Rep. 2019;6: 339–354. doi:10.1007/s40429-019-00267-x

4. Kaczorowski J, Bilodeau J, Orkin A, Dong K, Daoust R, Kestler A. Emergency Department–initiated Interventions for Patients With Opioid Use Disorder: A Systematic Review. Heard KJ, editor. Acad Emerg Med. 2020;27: 1173–1182. doi:10.1111/acem.14054

5. French R, Aronowitz SV, Brooks Carthon JM, Schmidt HD, Compton P. Interventions for hospitalized medical and surgical patients with opioid use disorder: A systematic review. Subst Abuse. 2022;43: 495–507. doi:10.1080/08897077.2021.1949663

6. Bahji A, Brothers TD, Mauer-Vakil D, Priest KC, Danilewitz M, Chopra N, et al. The Effectiveness of Inpatient Addiction Consult Services: A Systematic Review and Narrative Synthesis. Can J Addict. 2023;14: 9–19. doi:10.1097/CXA.0000000000000173
