# Supplementary material for: Interventions to improve access to opioid agonist therapy in acute hospitals: A scoping review
Source: PLOS Ment Health. 2025 May 30;2(5):e0000322. doi: 10.1371/journal.pmen.0000322 (PMC12798493; doi:10.1371/journal.pmen.0000322)
Supplement: Attachment — Submitted filename: response_letter.pdf [file pmen.0000322.s004.pdf]

Re: "Interventions to improve access to opioid agonist therapy in acute hospitals: a scoping review" (PMEN-D-25-00050)

Response to reviews and editor requests

26 March 2025

University College London, Gower Street, London WC1E 6BT
Tel: +44 (0)20 7679 2000
email@ucl.ac.uk
www.ucl.ac.uk

Journal Requirements:

Comment #1. Please provide separate figure files in .tif or .eps format

We have provided .tif formats, using the image tool available via the PLOS website.

University College London, Gower Street, London WC1E 6BT
Tel: +44 (0)20 7679 2000
email@ucl.ac.uk
www.ucl.ac.uk

Additional Editor Comments

Comment #2. *The reviewers have raised some valid points with respect to the ethics and robustness (primarily relating to the low response rate) of the survey component of your review, as well as its novelty, given the similar literature already published. Please pay particular attention to these concerns in your response to the reviewers comments, as this will help us assess this manuscript for suitability for publication.*

Many thanks for the opportunity to revise our article.

- In relation to ethics of the survey, this was specifically covered by an amendment to ethical approval of a wider research programme (please see comment #9, below).
- In relation to the robustness and low response rate, please also see comment #9 below. We should stress that the survey is not a core part of the review. While we feel it adds value and improves the review overall, we also understand its limitations and we are happy to take your guidance about its inclusion.
- In relation to the novelty of the review, please see comment #10 below. The existing reviews have related aims, such as interventions to improve medical care generally for people who use substances, but none focus on improving access to OAT in acute hospital settings.

Reviewer #1

Editorial issues

Comment #3. *Instead of a space between end of sentence and citation [#], there is a fullstop or comma. Might be just a file conversion issue.*

We have corrected the punctuation.

Comment #4. *Emergency Departments = make it lower case throughout*

Thank you – we have corrected this.

Comment #5. *They say "Two authors" did title and abstract screening but then say "(two of DL, MB, TB, MH, AH, VH, RG, KS, GW)" i.e. unclear who did what*

Each title and abstract was screened by two authors, with different pairs of authors screening each title and abstract. We have clarified this by saying that "DL, MB, TB, MH, AH, VH, RG, KS, GW contributed to this stage and two of these authors screened each article".

Substantive issues

Comment #6. *Overall I found the scoping review to be well done and well reported.*

Thank you!

Comment #7. *p. 5 "...known as 'patient directed discharge" – also include the more common previously accepted term "against medical advice"*

We have edited the text in the introduction to say: "patients may be aware of these issues and delay presentation at hospital, or leave hospital before treatment is complete (known as 'discharge before medical advice', 'patient-directed discharge', or 'discharge against medical advice')." When we refer to this situation later, we now use the term 'discharge before medically advised' following arguments that this term can be less stigmatising (Ann Intern Med. 2022;175:1761–1762. doi:10.7326/M22-2964).

Comment #8. *Could the search terms be put into a table or textbox instead of supplementary?*

We have put the full search terms for Medline in a box. To avoid duplation, we have retained full search terms for other databases in the Supplementary file rather than the main manuscript, as the terms as similar across databases.

Main issue

Comment #9. *I was taken aback by the inclusion of a questionnaire to corresponding authors, as I have not seen a scoping review have an add-on qualitative question component.*

This is an effort to give authors a voice and include their experiences of implementation and is not a core element of the review. We feel this element adds value to the review, even if it is unusual and does not follow formal qualitative methods. However, we do recognise that some

readers may feel this section is not sufficiently robust, and we are happy to follow PLOS editors' guidance around the value of this section, and whether it improves the article overall.

1) *I didn't see evidence of ethics approval for this qualitative component/study. This is a critical issue.*

Sorry that we did not provide this in the original submission. Ethical consideration for this survey was done prior to contacting authors via an amendment to the ethical approval for the wider research programme of which this review is one part (iHOST: Improving Hospital Opioid Substitution Therapy: https://fundingawards.nihr.ac.uk/award/NIHR133022). We have now included this information in the manuscript:

"Ethical approval for this questionnaire was provided on 14 August 2023 by LSHTM Observational Research Ethics Committee as part a wider research programme [Ref 27895]."

2) *While the authors say "All authors consented to publication of these responses" the process of consent is unclear.*

We have now clarified the process as follows: "We also asked respondents via email if they were willing for their answers to be published in a non-attributable format, and all authors agreed."

3) *The authors say that they described themes in the responses to these questions. However, they did not describe how they attended to rigour nor did they describe an analytical approach. These are critical components.*

We agree that this section of the review does not constitute a formal qualitative analysis. As we only have a small amount of data (brief answers to three questions from 16 authors), and a limited amount of space in which to present this information, we have simply highlighted common themes rather than attempted to add a qualitative analysis. As above, we do appreciate that some readers may feel this section is too informal and/or makes the review too complex, and we are happy to take guidance from PLOS editors regarding its inclusion.

4) *They indicate they received responses from 16/57 authors – which is a fairly poor response rate.*

A 'good' response rate is subjective, and we would highlight that we are not trying to make generalisable inferences from these survey responses. Nonetheless, 16/57 (28%) does not seem such a bad response rate, given we emailed the authors cold and many have moved into different roles and/or have clinical duties. Consider that the NSDUH response rate was 12% in 2022 and UK Biobank is 5%.

5) *Overall, the most significant issue (beside no ethics statement) is what does this qualitative component add?*

We feel this section adds some direct experience of implementation from people involved in the interventions. We highlight three themes that came up across respondents. In particular, respondents consistently mentioned the need to address stigma and perceptions that patients who use opioids may not be deserving of good quality care.

6) *I recommend submitting the qualitative component as a separate study.*

Our preference would be to keep these data together with the scoping review, because the authors' comments relate to the studies included in the review, and because the data collected in the brief questionnaire may not lend itself to a full study. However, as above we do appreciate there may be different views.

Reviewer #2

Comment #10. *The topic is important, and the paper is well written. That said, as the authors state, there are already four systematic reviews on similar topics, which somehow diminishes the novelty of the review.*

We agree that these existing reviews have some overlapping aims, but none focus on interventions that aim to improve access to OAT in acute hospitals. Where we list the four existing reviews (on page 19), we note that (a) focuses on general interventions in ED for people who use opioids – ie. some included studies are not related to OAT, while others are about OAT efficacy; (b) and (c) are reviews of studies aiming to improve general care medical care for patients who use opioids, and do not capture many of the studies in our review; and (d) focuses on addiction consult teams for patients using any substances. We have added the following text to highlight the novelty of this review:

"To our knowledge the present review is the first to focus on interventions that aim to improve access to OAT in acute hospital settings. Previous related reviews validate our findings that most evidence in this field is recent and from North America."

Comment #11. *Their inclusion of a survey to authors about the experience with implementation which could have added an interesting and different angle is limited by the fact that they got less than one third of responses.*

Please see our responses to comment #9 above.

Comment #12. *Another limitation of the present review, as the authors acknowledge, is that they only included articles written in English. This may have significantly biased their results towards only finding studies from North America. This is particularly problematic since as the authors also acknowledge studies from other settings where OAT coverage is higher (I.e., Western Europe) can provide some helpful insights to improve OAT coverage in North America. Another limitation of only including studies in English language is that probably most of QI projects wouldn't be translated to English and likely missed. Likewise, not searching for grey literature could also have resulted in having missed some key QI projects.*

We agree with this limitation, and also your additional point that the restriction to English language publications may have a greater impact on the inclusion of QI projects. We have added the following text to the discussion:

"Fifth, we may have disproportionately excluded evidence related to quality improvement projects for two reasons: (a) we focused on peer reviewed publications and evaluations of quality improvement projects may be published as grey literature; (b) evaluations of quality improvement projects may be less likely to be translated in English."

University College London, Gower Street, London WC1E 6BT
Tel: +44 (0)20 7679 2000
email@ucl.ac.uk
www.ucl.ac.uk
