# Supplementary material for: Interventions to improve access to opioid agonist therapy in acute hospitals: A scoping review
Source: PLOS Ment Health. 2025 May 30;2(5):e0000322. doi: 10.1371/journal.pmen.0000322 (PMC12798493; doi:10.1371/journal.pmen.0000322)
Supplement: Attachment — Submitted filename: response_letter_2.pdf [file pmen.0000322.s005.pdf]

Re: "Interventions to improve access to opioid agonist therapy in acute hospitals: a scoping review" (PMEN-D-25-00050)

Response to reviews and editor requests

14 April 2025

University College London, Gower Street, London WC1E 6BT
Tel: +44 (0)20 7679 2000
email@ucl.ac.uk
www.ucl.ac.uk

Additional Editor Comments

Comment #1. *I tend to agree with Reviewer 1 that in light of the limitations of the author responses to the questionnaire that it adds limited value to the rest of the review, so I suggest removing this from your submission.*

Thanks for the feedback and opportunity to revise our review. The author responses were not a central element of the review, and in light of feedback from you and the reviewers we have now removed this section.

Comment #2. *Please also address the other comments from Reviewer 1 so that we can consider your responses when making our decision about potential publication of your manuscript.*

Please see our responses to comments from Reviewer 1 below.

University College London, Gower Street, London WC1E 6BT
Tel: +44 (0)20 7679 2000
email@ucl.ac.uk
www.ucl.ac.uk

Reviewer #1

Comment #3. *Line 79: OAT [9]. And*

Many thanks - we have removed the full stop.

Comment #4. *pain control; AND (3) were*

Many thanks - we have added the word ‘and’.

Comment #5. *Line 136: "We had no restriction on the study design (we included both quantitative and qualitative studies) should read "We included both quantitative and qualitative designs"*

This sentence lists the criteria that were not restricted, including study design, date of publication, and country of intervention, so this edit would mean that the rest of the sentence wouldn’t make sense.

Comment #6. *Line 145: "We used the mixed methods appraisal tool (MMAT), which is an appraisal tool for reviews that..." should read "We used the mixed methods appraisal tool (MMAT) because of its utility for both qualitative and quantitative designs"*

Many thanks – we have made this edit.

Comment #7. *Line 161: "this list was" should read "COmponents included:*

Many thanks – we have made this edit.

Comment #8. *Line 165: drug use' AND (4)*

Many thanks - we have added the word ‘and’.

Comment #9. *Line 168: Very unclear sentence" We did not limit to interventions including these components and updated the list of components as we collected data."*

We have updated this sentence to read: “We labelled interventions according to these components, with interventions allowed to include more than one label. Where relevant interventions included components that did not appear on the list, we updated the list.”

Comment #10. *Line 122 "Citations were de-duplicated and uploaded to Covidence"; why were all articles not uploaded, then de-duplicated to demonstrate the management of duplicates?*

In this study, we deduplicated articles manually prior to uploading them to Covidence.

Comment #11. *I am unsure whether the MMAT is the best instrument to individually assess Qual and Quant designs, as MM focuses on integration*

Our understanding is that MMAT is designed for “as a checklist for concomitantly appraising and/or describing studies included in systematic mixed studies reviews” (http://mixedmethodsappraisaltoolpublic.pbworks.com/w/page/24607821/FrontPage, https://www.annualreviews.org/content/journals/10.1146/annurev-publhealth-032013-182440).

University College London, Gower Street, London WC1E 6BT
Tel: +44 (0)20 7679 2000
email@ucl.ac.uk
www.ucl.ac.uk

For future reference, we would be interested to know which tool might be more appropriate for this type of review and would be happy to note this in the limitations section of the discussion.

Comment #12. *I remain opposed to the inclusion of author responses. The qualitative component is biased by self-selection and response bias.*

This was not a central element of the review, and in light of your feedback we have now removed this section.
